# Artificial Neural Networks (ANN) for the Simultaneous Spectrophotometric Determination of Fluoxetine and Sertraline in Pharmaceutical Formulations and Biological Fluid

**Published:** 2017

**Authors:** Hamid Reza Akbari Hasanjani, Mahmoud Reza Sohrabi

**Affiliations:** a *Department of Chemistry, Faculty of Chemistry, Islamic Azad University, North Tehran Branch, Tehran, Iran.*

**Keywords:** Fluoxetine, Sertraline, Artificial Neural Networks (ANN), Spectrophotometric, Chemometrics

## Abstract

Simultaneous spectrophotometric estimation of Fluoxetine and Sertraline in tablets were performed using UV–Vis spectroscopic and Artificial Neural Networks (ANN). Absorption spectra of two components were recorded in 200–300 nm wavelengths region with an interval of 1 nm. The calibration models were thoroughly evaluated at several concentration levels using the spectra of synthetic binary mixture (prepared using orthogonal design). Three layers feed-forward neural networks using the back-propagation algorithm (B.P) has been employed for building and testing models. Several parameters such as the number of neurons in the hidden layer, learning rate and the number of epochs were optimized. The Relative Standard Deviation (RSD) for each component in real sample was calculated as 1.06 and 1.33 for Fluoxetine and Sertraline, respectively. The results showed a very good agreement between true values and predicted concentration values. The proposed procedure is a simple, precise and convenient method for the determination of Fluoxetine and Sertraline in commercial tablets.

## Introduction

**Figure 1:**
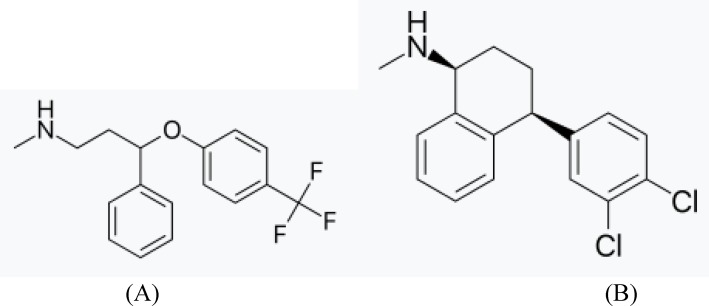
The structures of fluoxetine HCl (A) and sertraline HCl (B).

Fluoxetine HCl (FLX) is an antidepressant of the selective serotonin reuptake inhibitor (SSRI) class. It is chemically designated as N-methyl- 3-phenyl-3-(4-(trifluoromethyl)phenoxy) propan-1-amine, (Figure 1A).

It is used for the treatment of depression. Being one of SSRI drugs, it acts by increasing the extracellular level of the neurotransmitter serotonin by inhibiting its reuptake into the cell (1). Fluoxetine is used in the treatment of major depression (including pediatric depression), panic disorders and premenstrual dysphonic disorder. It’s also been used for cataplexy, obesity and alcohol dependence (2).

Sertraline ((1S,4S)N-methyl-4-(3,4-dichloro phenyl)- 1,2,3,4-tetrahydro-1-naphthylamine, SER) (Figure 1B) , categorized as a second generation antidepressant drug belongs to the SSRI class. It is approved by the USFDA for the treatment of depression, obsessive–compulsive disorder, posttraumatic stress disorder, social anxiety disorder, postmenopausal dysphoric disorder and panic disorder (3). 

Simultaneous determination of components in multi components drug formulations could be a difficult task, especially when the absorption spectra of these components have strong overlapping, which prevents UV–Vis spectrometric determination of these components. Due to the fact that improvements happened in field of computer technology, several methods with great potential have been introduced. They have been successfully applied to many difficult search and optimization problems in a diversity of research domains, including, economy (4), risk management (5, 6), business (7), medicine (8, 9) and chemistry (10, 11)

**Figure 2:**
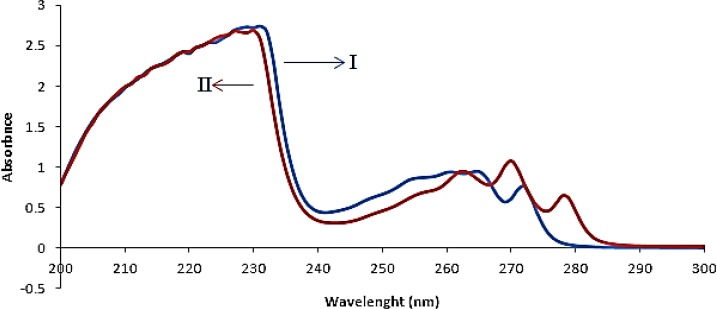
Absorbance spectra of fluoxetine 20 𝜇𝑔𝑚𝐿−1 (I) and sertraline 20 𝜇𝑔𝑚𝐿−1(II) in ethanol

**Figure 3:**
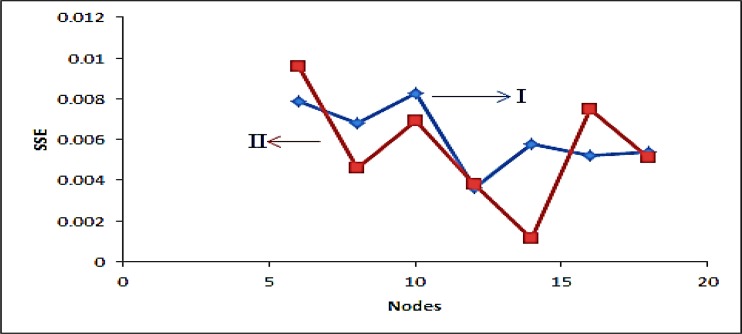
The relationship between numbers of nodes in the hidden layer versus SSE for Fluoxetine (I) and Sertraline (II

**Figure 4:**
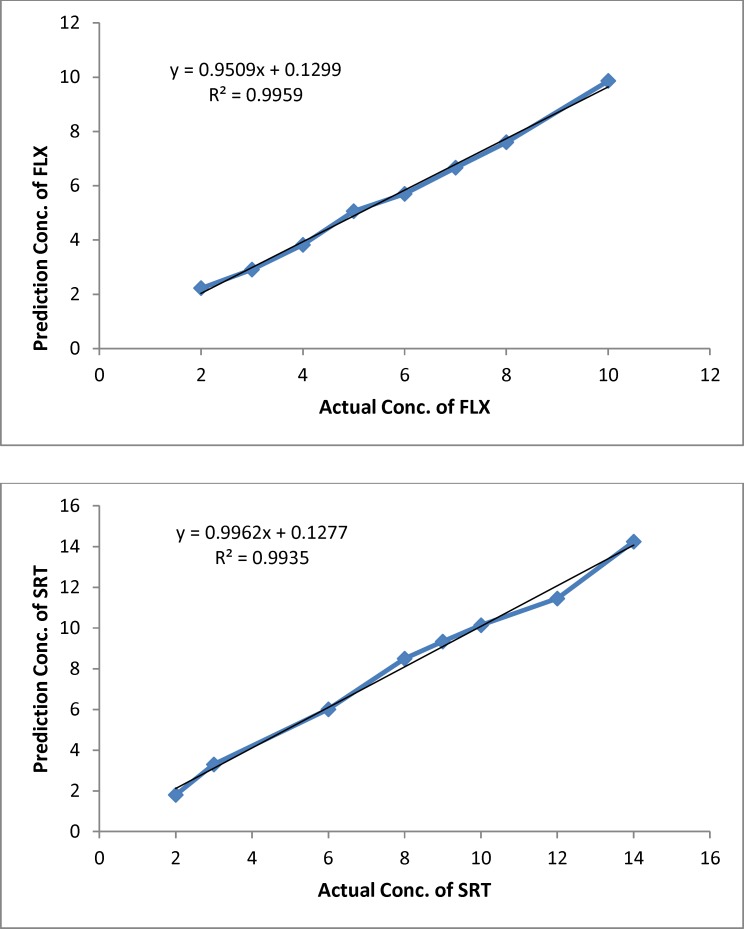
Plots of predicted concentration versus actual concentration for Fluoxetine (FLX) and Sertraline (SRT) by ANN (μgmL−1

**Figure 5:**
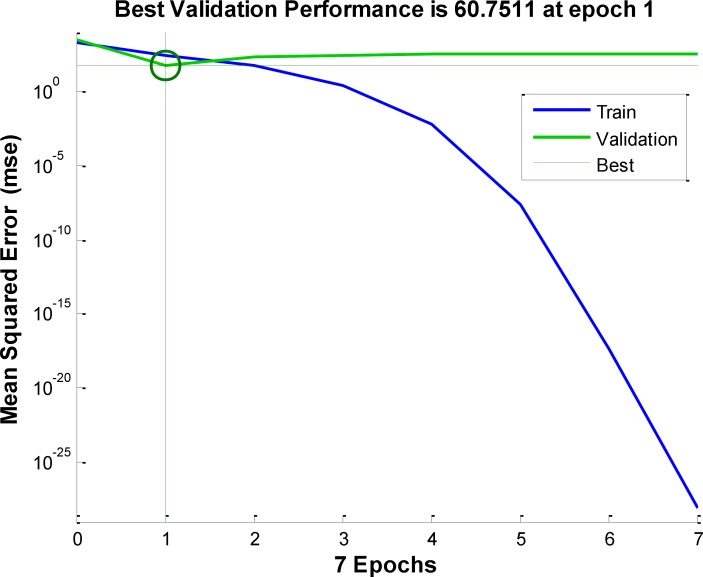
Error performance for tarining of ANN with actual inputs

**Fig.6:**
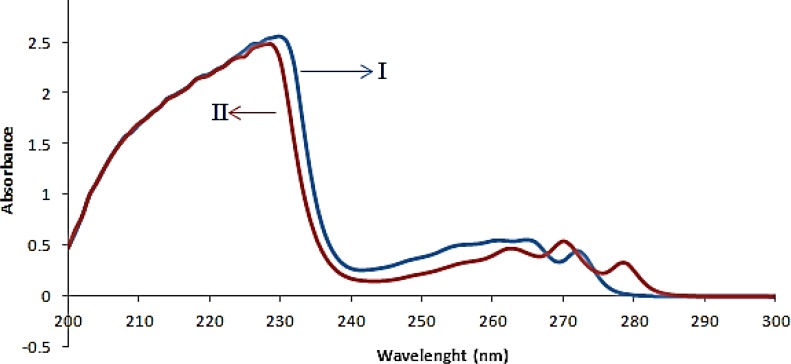
Absorbance spectra of fluoxetine 20${\mathrm{\mu gmL}}^{-1}$ (I) and sertraline 20${\mathrm{\mu gmL}}^{-1}$(II) in ethanol

**Table 1:** Composition of the calibration sample

**Sample**	**Concentration (** ${\mathrm{\mu gmL}}^{-1}$ **)**	
	**FLX**	**SRT**
1	2	1
2	2	4
3	2	6
4	2	8
5	2	0
6	4	2
7	4	0
8	4	8
9	4	6
10	4	4
11	6	1
12	6	4
13	6	8
14	6	0
15	6	2
16	8	6
17	8	4
18	8	0
19	8	3
20	8	8

**Table 2:** Composition of validation set, their prediction by the ANN model and statistical parameters for the system.

**Sample**	**Actual**		**Prediction**		**Recoveries (%)**	
	**FLX**	**SRT**	**FLX**	**SRT**	**FLX**	**SRT**
1	2	2	2.20	1.80	110.00	90.00
2	3	3	2.91	3.30	97.00	110.00
3	4	6	3.82	6.01	95.50	100.16
4	5	8	5.06	8.50	101.20	106.25
5	6	9	5.70	9.34	95.00	103.77
6	7	10	6.66	10.14	95.14	101.40
7	8	12	7.60	11.45	95.00	95.41
8	10	14	9.86	14.24	98.60	101.41
Mean Recovery (%)					98.43	101.08
RMSE					0.24	0.33

**Table 3:** Determination of Fluoxetine and Sertraline by using ANN model in pharmaceutical formulation

**Sample**	**FLX**		**SRT**	
	**Actual**	**Prediction**	**Actual**	**Prediction**
1	20	19.31	100	101.17
2	20	21.22	100	99.49
3	20	19.44	100	98.62
Mean Recovery (%)	99.95		99.76	
RMSE	0.29		0.42	
RSD	1.06		1.33	

**Table 4:** Determination of fluoxetine and sertraline in urine using ANN model .

**Sample**	**Added (** ${\mathrm{\mu gmL}}^{-1}$ **)**	**Found (** ${\mathrm{\mu gmL}}^{-1}$ **)**	**Recovery (%)**
Urine sample 1	1.50	1.42	94.7
Urine sample 2	3	3.31	94.6

**Table 5:** The ANOVA results by applying the proposed method to the real sample

***Source of Variation***	***SS***	***df***	***MS***	***F***	***P-value***	***F crit***
Between Groups						
Fluoxetine	0.1089	1	0.1089	0.137483	0.746386	18.51282
Sertraline	0.893025	1	0.893025	4.719382	0.161935	18.51282
Within Groups						
Fluoxetine	1.5842	2	0.7921			
Sertraline	0.37845	2	0.189225			
Total						
Fluoxetine	1.6931	3				
Sertraline	1.271475	3				

ss, sum of squares; df, degree of freedom; MS, mean squares.

Numerous analytical methods have been developed for the determination of fluoxetine and sertraline in biological fluids; the methods published before 1996 were reviewed by Eap and Baumann (12). These methods use liquid chromatography (LC; 13–21), micellar electrokinetic capillary chromatography (22), gas chromatography (23–29), capillary electrophoresis (30–31), and immunoassay (32). They offer the required sensitivity and selectivity for the determination of the investigated drugs and/or their metabolites in biological fluids; however, their sophisticated instrumentation and high analytical cost limit their use in quality-control laboratories for determination of these drugs in their pharmaceutical dosage forms. The analytical techniques reported for the determination of fluoxetine, sertraline, and paroxetine in pharmaceutical dosage forms include trimetry (33, 34), voltammetry (35, 36), LC (37–40), capillary electrophoresis (41), and spectrophotometry (42–49). The titrimetric methods are time consuming, and they lack the necessary sensitivity. The voltammetric, chromatographic, and electrophoretic methods use dedicated and/or expensive instruments that are not available in most quality-control laboratories. In general, spectrophotometry is considered the most convenient analytical technique because of its inherent simplicity, low cost, and wide availability in most quality-control laboratories. However, the spectrophotometric methods reported for the determination of fluoxetine and sertraline in their pharmaceutical formulations are associated with some drawbacks such as decreased selectivity due to measurement in the ultraviolet region (42, 43) and/or decreased simplicity of the assay procedure (e.g., laborious extraction steps in ion-pair formation-based methods;(44–46). 

Developing new alternative chemometric methods is one of the open issues in the area of analytical chemistry. New combined mathematical techniques provide in some cases more accurate results than those obtained by classical methods (50-54).

Advanced statistical methods have allowed many experimentalists to retrieve qualitative and quantitative information from data sets which is not evident otherwise. The application of statistical methods in spectroscopic analysis has been growing rapidly in recent years, but it is mainly limited to classical chemometrics at the moment.

Here, we report our investigation of an approach in which an alternative approach based on application of Artificial Neural Network (ANN) designed to decrease the present overlap. ANN is a computer algorithm whose structure and function came from the structure and learning behavior of biological neurons. This algorithm is typically employed to classify a set of patterns into one of several classes. The classification rules are not written into the algorithm, but are learned by the network from examples. The basic elements of ANN are processing elements (Pes) and weighted connections. The collection of processing elements defined as a layer includes the input, one or more hidden layers, and an output layer. Each processing element receives values from all its input connections, performs a previously defined mathematical operation and produces a single output value. The connection weights store the information in the form of weight matrices (55, 56). The value of the connection weights is determined by the neural network learning procedure. Learning therefore is the most appealing quality of ANN which could be either ‘‘supervised’’, where sample input–output pairs are presented or ‘‘unsupervised’’, where the network organizes itself. The most successful algorithm in solving different problems so far has been the back propagation learning method. In this method, the partial derivative of an error criterion with respect to the weights in turn is adjusted as the negative gradient to minimize the error function (57).The aim of this study was to apply the ANN with back propagation learning algorithm to resolve the overlapped UV–Vis absorption spectra and to quantify these components simultaneously. This method was applied for the analysis of Fluoxetine and Sertraline in Pharmaceutical Formulation.

*Methodology*

*Artificial neural networks*

ANN is a simulation of a real neurons system that contains a collection of neuron units communicating with each other via axon connections. Each neuron contains input, weights associate with each input, transfer function and output (58). The Back-Propagation algorithm (B.P.) is perhaps the most widely used supervised training algorithm in multivariate calibration. The network constructed input, hidden and output nodes in three layers.

*Data processing*

The commonly used method for estimating the generalized error in neural network is cross-validation. In this method the calibration set is randomly divided into two subsets, one used for training (including 70% of the calibration samples), and the other for testing (the remaining samples). The test set is held out during training, which avoids the overlap between training data and test data, yielding a more accurate estimate for generalization performance of the algorithm (59, 60).

In order to perform a supervised training and prediction we need a way of evaluating the ANN output. The most commonly used stopping criterion in neural network training is the Sum-Square-Errors (SSE), calculated for the training subsets as:

$\mathrm{SSE}=\frac{1}{N}\sum_{p=1}^{N}\sum_{i=1}^{M}{\left(o_{\mathrm{pi}}-t_{\mathrm{pi}}\right)}^{2}$

where o_pi_ and t_pi_ are respectively actual and target solution of the ith output nodes on the pth example, N the number of training examples and M is the number of output nodes (61, 62). The aim of any training set is to reach the smallest SSE value possible in the shortest possible time while avoiding the overtraining problem.

 In this work, sigmoid transfer function was applied between the input layer and output node as:

## Experimental

*Apparatus and software*

A Shimadzu uv-2100 double-beam UV–Vis spectrophotometer with a 1-cm path length quartz cell was utilized. Calculations and the signal transforms were performed in EXCEL and MATLAB 7.1 environment. The spectrophotometric measurements were carried out at room temperature (mean of about 20 ℃) and all solutions were prepared on the same day before that of the analysis.

A model pHs-3C pH meter was calibrated by using buffer solutions and then used for pH measurements. The pH of the solutions was surveyed in the range of pH 2.0 – 9.0.

*Reagent and chemicals*

All chemicals and solvents were of analytical reagent grade.

Pure Fluoxetine and Sertraline drugs and its pharmaceutical dosage form containing 20 mg of FLX and 100 mg of SRT were donated by Dr. Abidi Company (Tehran – Iran). Sulfuric acid and NaOH were purchased from Merck (Darmstadt, Germany).Company.

*Standard Solutions*

Stock standard solutions of fluoxetine and sertraline were prepared by dissolving 20.0 mg of fluoxetine and 20.0 mg of sertraline in ethanol in 100 mL volumetric flaks and diluted to the mark with the solvent. 

Working solutions were prepared by appropriate dilution of the stock solution in ethanol to reach concentration ranges of 2-8μg *mL*^-1^and 0-8μg *mL*^-1^for fluoxetine and sertraline, respectively.

*Preparation of real samples*

Ten tablets were finely powdered and an appropriate portion (equivalent to the median mass of one tablet) was dissolved in 100 mL of ethanol, It was mechanically shaken for a period of 20 min and filtered into a 250 mL calibrated flask. The residue was washed twice with the same solvent and diluted to the volume. After filtration, the obtained clear solution was adjusted to the volume of 100 mL with the same solvent. This solution was further diluted to get the suitable concentration for the UV measurements (33).

*Analysis of urine samples*

Urine spiked with fluoxetine and sertraline was obtained by following procedure; an aliquot of pure fluoxetine and sertraline was added into 10 mL urine sample. A 1 mL of the resulting urine solution was mixed with 5 mL (0.2 M) sodium carbonate buffer and 10 mL butyl chloride. The mixture was rotated for 20 min and centrifuged at 2500 rpm for 10 min. The butyl chloride layer was separated and then evaporated till dryness. Resultant residue was dissolved in universal buffer (at different pH) into a 10 mL volumetric flask and diluted to the mark with buffer solution.

## Result and Discussion

*Design of mixture*

The absorption spectra of FLX and SRT overlapped clearly, as depicted in Figure 2. This simply indicated that simultaneous determination of two components by direct UV–Vis spectrophotometry could not produce reliable result for quantitative analysis of binary mixture of FLX and SRT.

The multivariate calibration requires a suitable experimental design of the standard composition of calibration set to provide the best prediction. The orthogonal array design method was used to construct the calibration set. Application of four-level orthogonal array design led to construction and optimization of the ANN model. Thus, two binary set of synthetic mixtures present in random ratio were prepared, one set with 20 samples for construction and optimization of the ANN model (Table 1), and one set with 8 samples were employed as an independent test to evaluate the quality of the model.

*Effect of pH*

The effect of pH was studied for the separation of spectral. The pH changes of the samples had no effect in separation or reduction of overlapping spectra. Due to their mutual interference, simultaneous determination of the binary mixture of FLX and SRT is not possible by using classical spectrophotometric method.

*Training and optimization of ANN models*

The calibration data obtained from experimental were gathered in a matrix data by Microsoft Office Excel (Ver. 2007) and transferred to MATLAB (Ver. 7.1). A local written program in MATLAB environment was used to establish the BP-ANN with sigmoidal transformation function in the nodes. The network involved three layers, namely, single input layer with 31 input nodes, which represent the absorbance intensities measured at 31 different wavelengths from each spectrum. Hidden layer was consisted of 6 optimized process elements and output layer with a single output node which contained the concentration of component. Although, a neural network has the property to model multiple responses simultaneously, it is recommended that one model only generate one response at a time and therefore our network had a single output node. Network optimization was performed by changing each time one of the internal parameters of ANN such as the number of nodes in the hidden layer, number of epochs and learning rate while keeping rest of them constant.

The proper number of nodes in the hidden layer was determined by training ANN with different number of nodes in hidden layer, it has a great effect on the prediction result. Figure 3 shows the variation of SSE value of the network when the number of node in hidden layer changed in the range of 2–18 neurons. The result shows that, by increasing the number of nodes from 2 to 6 and 2 to 12 for FLX and SRT respectively, the SSE value decreasing and then increasing rapidly. Based on data in Fig. 3, a minimum in SSE occurred when 6 and 12 nodes were used in hidden layer of FLX and SRT, respectively. To study the ability of established ANN in prediction of individual analysis found in synthetic and real mixtures, eight binary synthetic mixture test samples were analyzed using proposed method. The predicted concentration of the analytes in each sample was then compared with the known concentration of the analytes in the respective sample; the predicted results are given in Table 2. The plots of the predicted concentration versus actual values are shown in Fig. 4 for Fluoxetine and Sertraline, as well as the line equations and R^2 ^values.

Performances of these neural network is compared by their generalization accuracy and convergence speed. Figure 5 show the error versus number of iteration for training of neural network.

As in the above descriptions, the reliable results for the quantitative prediction of FLX and SRT in samples were obtained by the application of ANN approach. 

*Result for real sample*

By using the prepared tablet solutions explained in the above section “Preparation of real samples”, ANN were applied to the simultaneous quantitative determination of FLX and SRT in tablets. The obtained experimental results of two drugs in marketed tablets were shown in Table 3. Moreover, a comparison of the spectra from the FLX and SRT in standard and drug formulation solutions shows similar spectral patterns (Figure 6).

The statistical parameters, namely, percent relative standard deviation, root mean square error are shown in Table 3.

*Determination of fluoxetine and sertraline in pharmaceutical formulations and biological fluids*

In order to show the analytical applicability of the proposed methods, first calibration curve obtained from ANN model at PH 2 were applied to determination of fluoxetine and sertraline in real samples (pharmaceutical formulations) and complex matrices, i.e. urine . The results showed that satisfactory recovery for fluoxetine and sertraline could be obtained (Tables 4) using the recommended procedures. Results of the determination are summarized in Table 4. The data obtained by these methods reveal the capability of the methods for determination of fluoxetine and sertraline in real samples such as pharmaceutical formulations and complex matrices such as urine without considerable error. The average recoveries in pharmaceutical formulations (Dr abidi Tablets) and complex matrix (urine) are summarized in Table 4.

*ANOVA*

The results achieved by the proposed method were compared with using one-way ANOVA test. The calculated F-values (P = 0.05) were less than the tabulated F-values. No significant differences were observed between the results of the proposed method at 95% confidence level. The corresponding results are summarized in Table 5. Findings of our study suggest that, there are no significant errors in simultaneous determination of FLX and SRT by proposed methods.

## Conclusion

This study confirmed the high potential of usefulness for ANN methods combined with UV–Vis spectrophotometer for simultaneous quantitative analysis of two components Fluoxetine and Sertraline in pharmaceutical formulation.

Chemometrics calibration techniques in spectral analysis have been widely used in quality control of drugs in mixture and multicomponent formulations with overlapping spectra, as separation procedures in the drug determinations are not required.

Although other methods such as chromatographic methods can be used to determine these components in pharmaceuticals, they are both more time consuming and expensive than the procedure here developed.

The results obtained in this paper encourage us to apply the proposed technique for the simultaneous determination of FLX and SRT in tablets without making use of any pretreatment procedure. 
